# Bathometer: lightning fast depth-of-reads query

**DOI:** 10.1093/bioinformatics/btab372

**Published:** 2021-05-13

**Authors:** U Stenzel, S Horn

**Affiliations:** Department for Molecular Biochemistry, Rudolf Schönheimer Institute of Biochemistry, University of Leipzig, 04103 Leipzig, Germany; Department for Molecular Biochemistry, Rudolf Schönheimer Institute of Biochemistry, University of Leipzig, 04103 Leipzig, Germany; West German Cancer Center, Department of Dermatology, University Hospital Essen, 45122 Essen, Germany; German Cancer Consortium (DKTK), University Hospital Essen, 45122 Essen, Germany

## Abstract

**Motivation:**

The query for the number of reads overlapping a given region is a common step in the analysis of Illumina sequencing data. Sometimes, these queries are not conveniently precomputable. It seems beneficial to make this kind of arbitrary query as fast and convenient as possible.

**Results:**

We present Bathometer, a tool that indexes BAM files in a space efficient way, which allows ad hoc queries for the number of reads overlapping any given genomic region to be answered much more quickly than by counting with common tools such as Samtools, while incurring much less disk I/O.

**Availabilityand implementation:**

Bathometer is implemented in C, licensed under the GNU General Public License version 3+ and freely downloadable from Bitbucket (https://bitbucket.org/ustenzel/bathometer)

**Supplementary information:**

Supplementary data are available at *Bioinformatics* online.

## 1 Features

To use Bathometer, one or more BAM files have to be indexed by running bathometer index. The index, which has a size of approximately 5% that of the input file, is by itself sufficient to answer queries. The input does not need to be sorted, and, at least in principle, does not even need to be in BAM format. This is in contrast to Samtools (Li *et al.*, 2009), which needs both an index and the original, sorted BAM file.

Queries are run against one or more indices using bathometer query or bathometer fpkm. The former counts reads overlapping query regions, while the latter additionally normalizes these counts and reports FPKM (Fragments Per Kilobase and Million reads mapped), which is customary in the analysis of RNA sequencing experiments. Queries can be in the common file formats BED or GFF, or can be specified on the command line. In either case, Bathometer repeats the queries to its standard output, and adds the results in additional columns, thus directly producing a matrix with one sample per column and one query per row.

It should be noted that Bathometer effectively counts reads when creating the index. Consequently, any filters have to be applied at the index creation stage, and further filtering at the query stage cannot be supported.

## 2 Data structures

Bathometer aims for an index that is compact and can be used without having to be read into memory completely. An index stores for each strand of each reference sequence the list of starting positions and the list of end positions of all reads.

The lists of positions are both increasing lists of integers, and are stored in Elias-Fano coding (Elias, 1972). Briefly, this coding splits each position into a low part and a high part, where the low part is stored explicitly in binary, forming a vector *L*, while for the high part, differences between consecutive entries are stored in unary, forming the bitvector *H*. The split is chosen in such a way that approximately half the bits in *H* are set.

To compute the number of reads overlapping interval (*a*, *b*), Bathometer subtracts the number of reads ending before *a* from the number of reads starting before *b*. This requires it to find the length of the shortest prefix of *H* that contains a given number *n* of set bits (usually denoted ${\text{select}}_{1}(H,n)$). This is supported in practically constant time by the ‘simple’ select method of Vigna (2008): *H* is augmented with an inventory table that contains the prefix lengths for round numbers. select is realized by a lookup in the inventory, followed by scanning a very limited stretch of *H* from the indicated position.

The Elias-Fano coding has a size very close to the information-theoric limit. Thus, the Bathometer index is very nearly the smallest index that can conceivably support range queries. Furthermore, queries need to access a miniscule portion of the index: two entries of each inventory, a few words of each *H* vector and one entry of each *L* vector. With the index already in RAM, this incurs only six cache misses and with the index residing on secondary storage, the operating system will typically have to read six pages of 4kB each into RAM. On today’s common computers, a substantial number of indices could realistically remain in RAM concurrently. In addition, Bathometer has to parse some metainformation once per query, but this overhead is negligible for batch queries.

In contrast, any program that aims to answer similar queries using the standard BAM index, will have to parse the whole index, and the header of the BAM file at least once per invocation. Then for every query, multiple ‘bins’ have to be looked up in the index structure, and pieces of the BAM file have to be loaded, decompressed and parsed for each non-empty bin. This necessarily involves both more disk I/O and more processing, and it requires more space (either RAM or disk) to keep both the index and the raw data available.

## 3 Performance comparison

We compared the performance of bathometer query to running samtools view -c -L, which produces useless output, but executes approximately the equivalent query. Table 1 lists the time and memory requirements to build the index (the needed disk space consists of both the index and the raw data for Samtools, but only the index for Bathometer), Table 2 lists the times to run the queries. The test data result from RNA sequencing of melanoma tumors of 91 patients (Gide et al., 2019), available under the accession PRJEB23709 at ENA (the *Gide* dataset) and approximately 8 GB of whole genome shotgun sequences of wheat, available under the accessions SRR12687020 and SRR12687022 at ERA (the *Wheat* dataset). Sequencing reads were mapped to the human reference GRCh38 or the Wheat reference genome 1.30 using Hisat2. The query is either a BED file containing 5818 regions, see Supplementary Data, or the genome annotation for wheat as obtained from NCBI.

**Table 1.** Resources needed to index two datasets

	User	System	Wallclock	Memory	Disk
Bathometer (Gide)	4 h 17 min	3 min	4 h 40 min	1.36 GB	21.2 GB
Samtools (Gide)	1 h 33 min	3 min	2 h 37 min	0.24 GB	701 GB
Bathometer (Wheat)	2 min 16 s	1.9 s	3 min 5 s	516 MB	0.37 GB
Samtools (Wheat)	44 s	1.2 s	49 s	80 MB	5.73 GB

**Table 2.** Time to query two datasets

	User	System	Wallclock
Bathometer *(Gide)*, disk	1 s	4.5 s	3 min
Bathometer *(Gide)*, cache	1.7 s	8.1 s	1 min 3 s
Bathometer *(Gide)*, ssd	0.5 s	0.7 s	1.2 s
Samtools *(Gide)*, disk	1 h 58 min	2 min	3 h 23 min
Samtools *(Gide)*, cache	1 h 59 min	2 min	2 h 17 min
Samtools *(Gide)*, ssd	11 min	23 s	12 min
Bathometer *(Wheat)*, disk	0.23 s	0.03 s	0.54 s
Bathometer *(Wheat)*, cache	0.28 s	0.02 s	0.20 s
Bathometer *(Wheat)*, ssd	0.17 s	0.03 s	0.21 s
Samtools *(Wheat)*, disk	50 s	1.5 s	1 min 6 s
Samtools *(Wheat)*, cache	46 s	1.0 s	47 s
Samtools *(Wheat)*, ssd	46 s	1.4 s	48 s

All tests were run on an Intel(R) Xeon(R) Gold 6244 with 384 MB of RAM under Linux. The data resided on a distributed Ceph filesystem backed by hard drives (‘disk’), or on a local SSD (‘ssd’), or in the buffer cache (‘cache’) after running the same query twice. Lacking a sufficiently large SSD for the full dataset, we ran the Samtools *Gide* tests on the first seven patients and extrapolated to the full dataset.

## Funding

This work was partly funded by the Deutsche Forschungsgemeinschaft (DFG, German Research Foundation; HO 6389/2-1 [KFO 337], 6389/3-1 [Cryptamel]).

*Conflict of Interest*: none declared.
